# Mounting antiscience aggression in the United States

**DOI:** 10.1371/journal.pbio.3001369

**Published:** 2021-07-28

**Authors:** Peter J. Hotez

**Affiliations:** 1 Texas Children’s Center for Vaccine Development, Departments of Pediatrics, Molecular Virology & Microbiology, and Center for Health Policy, National School of Tropical Medicine, Baylor College of Medicine, Houston, Texas, United States of America; 2 Department of Biology, Baylor University, Waco, Texas, United States of America; 3 Hagler Institute of Advanced Study and Scowcroft Institute of International Affairs, Texas A&M University, College Station, Texas, United States of America; 4 Center for Health and Biosciences, James A Baker III Institute for Public Policy, Rice University, Houston, Texas, United States of America

## Abstract

This Perspective article discusses a troubling new expansion of anti-science aggression in the United States which arises from far-right extremism, including some elected members of the US Congress and conservative news outlets that target prominent biological scientists fighting the COVID-19 pandemic.

## Teaser

There is a troubling new expansion of antiscience aggression in the United States. It’s arising from far-right extremism, including some elected members of the US Congress and conservative news outlets that target prominent biological scientists fighting the COVID-19 pandemic.

A band of ultraconservative members of the US Congress and other public officials with far-right leanings are waging organized and seemingly well-coordinated attacks against prominent US biological scientists. In parallel, conservative news outlets repeatedly and purposefully promote disinformation designed to portray key American scientists as enemies. As a consequence, many of us receive threats via email and on social media, while some are stalked at home, to create an unprecedented culture of antiscience intimidation.

Over the spring and summer of 2021, four major incidents stand out. First, Georgia Rep. Marjorie Taylor Greene (R-GA) introduced house bill 2316 [1]. The “Fire Fauci Act” called for halting payment of Dr. Anthony Fauci’s salary as Director of the National Institute of Allergy and Infectious Diseases, and auditing his digital correspondence and financial transactions. Greene’s follow-up press conference on 21 June 2021 included 13 Republican House supporters or co-sponsors, possibly the largest congressional delegation in modern times to single out and attempt to humiliate a prominent American scientist.

Also in June, the Republicans organized a House Select Subcommittee on the origins of COVID-19 with the presumption that it was ignited by gain-of-function genetic engineering research from the Wuhan Institute of Virology. Despite evidence pointing to spillover from a viral infection in bats to additional mammals and ultimately humans accounting for previous coronavirus epidemics [2], the hearings took on a sinister tone, pointing fingers at virologists both in the US and China. Rep. Jim Jordan (R-OH), stated that Dr. Fauci was "afraid of something" and falsely claimed that he was covering up the engineering of the SARS-CoV-2 coronavirus [3]. The far-right media harasses or stalks other prominent US scientists, including Dr. Peter Daszak who heads the EcoHealth Alliance and conducts research on the zoonotic origins of human virus infections [1].

Vaccines and vaccine scientists are also targeted. Alongside the June Republican COVID-19 origins hearings, Senator Ron Johnson (R-WI) organized a roundtable in Milwaukee to highlight the rare adverse side effects from COVID-19 vaccines [4], as evening Fox News anchors promoted fake claims regarding deaths from COVID-19 vaccinations [5]. In July, Rep. Greene declared on Twitter that a COVID-19 vaccine is “a political tool used to control people”, while Rep. Madison Cawthorn (R-NC) said that door-to-door COVID-19 vaccinations were just a step away from US Government confiscations of guns and bibles, and Rep. Lauren Boebert (R-CO) referred to vaccinators as “needle Nazis”. Days later, the medical director for vaccines in the Tennessee Department of Health was abruptly terminated for her efforts to vaccinate minors (14 and up) without parental consent. These actions were concurrent with state efforts to halt vaccine advocacy and outreach to teens and adolescents, and at a time when the delta variant is accelerating [6]. As a vaccine scientist and author of a book explaining why autism, including my adult daughter’s autism, is unrelated to vaccines [7], I am also a target of antivaccine activists, including those writing menacingly about “patriots” who will seek me out. During a June 2021 interview with the staunchly conservative Florida Governor, a Fox News anchor referred to me as “infamous”, and “notorious” [8].

These events have context. Prior to 2021, a program of antiscience disinformation that dismissed the severity of the COVID-19 pandemic was aggressively pursued by a White House committed to policies of “America First”. The America First element of the far right focuses on nativism, anti-immigration, and a foreign policy built around strong military build-up and deterrence, and confrontation with China. A darker view links it to voter suppression, and loyalty tests to the former President that question the veracity of the 2020 Presidential election. Harvard University political scientist, Steven Levitsky (the co-author of *How Democracies Die*), point out how these elements converge to form a modern day authoritarian regime [9], seeking to concentrate power among a selected few while limiting the reach of opposition groups.

Historically, such regimes viewed scientists as enemies of the state. In his 1941 essay, *Science in the Totalitarian State* [10], Waldemar Kaempffert outlines details using the examples of Nazism under Hitler, Fascism under Mussolini, and Marxism and Leninism [10]. For example, under Stalin, the study of genetics and relativity physics were treated as dangerous western theories, and potentially in conflict with official social philosophies of state [11]. Today, there remain examples of authoritarian regimes that hold similar views. In 2019, the Hungarian Government under Prime Minister Viktor Orbán took over the control of the Hungarian Academy of Scientists. Brazil’s President Jair Bolsonaro cut funding for Brazilian scientific institutions and universities while downplaying the severity of the COVID-19 pandemic or undermining evidence of deforestation in the Amazon due to climate change.

Such activities are sometimes conducted under an alternative or replacement intellectual framework. In the book *Twilight of Democracy*: *The Seductive Lure of Authoritarianism*, Anne Applebaum illustrates the rationale for authoritarian targeting, and ultimately replacement, of the intelligentsia, including the scientists [12]. She cites the work of Julien Benda, a French essayist who in his 1927 book, *The Betrayal of the Intellectuals* (in French, *La trahison de clercs*), identifies the need to establish a core element of intellectuals to legitimize the authoritarian regime. To dismantle a legitimate scientific infrastructure it becomes essential to create an alternative narrative. For that reason Orbán does not close the Hungarian Academy of Sciences, but seizes its control. In the 1930s and 40s, Stalin replaced Vavilov with Trofim Lysenko and his pseudoscientific theories of vernalization [11]. Along similar lines, the rise of antiscience in an authoritarian America is notable for its intellectual cover. Experts affiliated with far right-leaning think tanks have adopted positions on herd immunity, vaccinations, and other COVID-19 prevention approaches that fit the America First narrative. In some cases these views are reinforced by intellectuals on the dark web.

**Fig 1:**
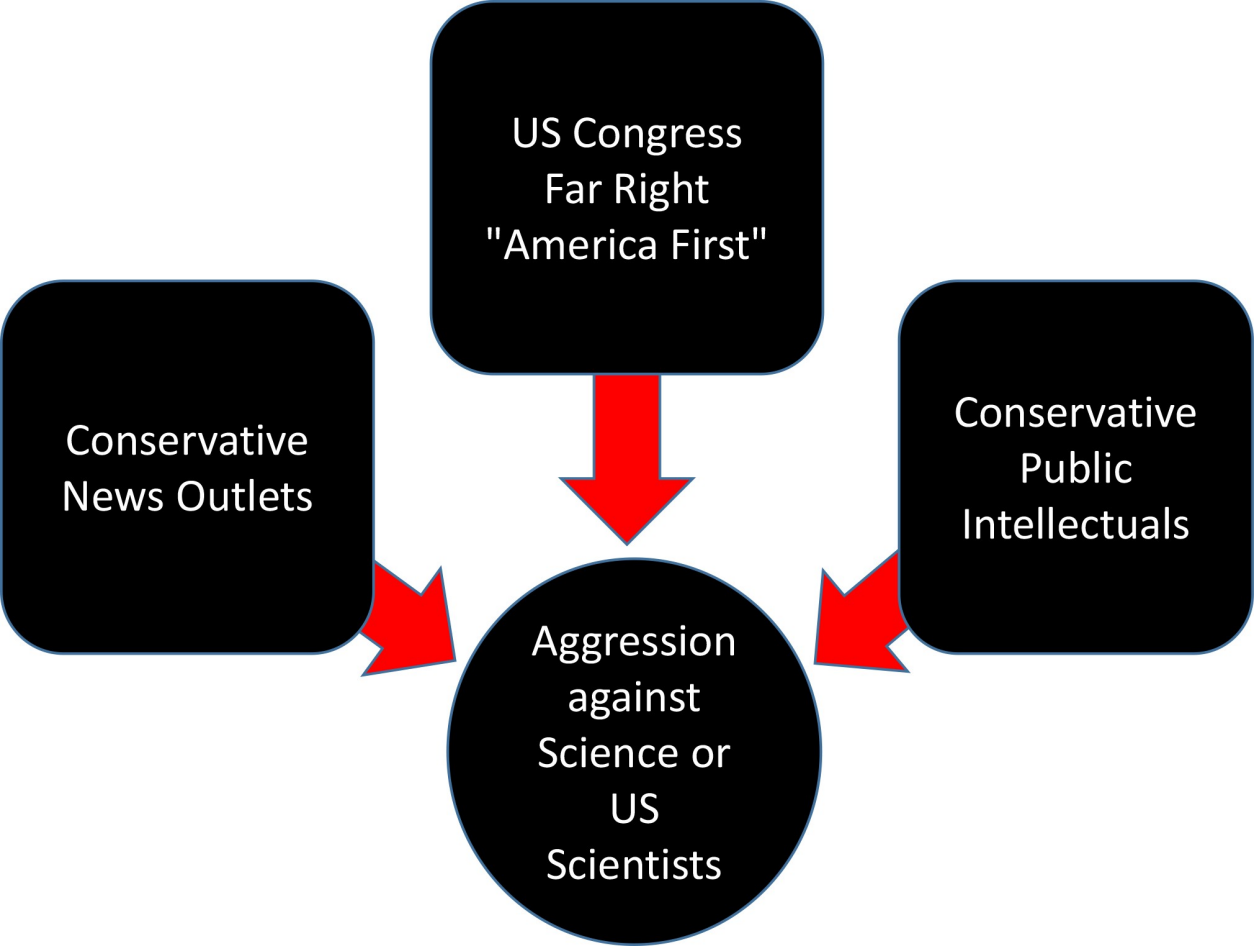
Aggression against science or US scientists in the Year 2021.

In summary, the aggression against science and scientists in America arises from three sources: 1) Far-right members of the US Congress, 2) the conservative news outlets and 3) a group of thought leaders who provide intellectual underpinnings to fuel the first two elements (Fig 1).

For researchers working in the pandemic response to continue to do so effectively, we seek help in halting the aggression. This is essential not only for our personal safety or national security, but also the reality that attacking science and scientists will both promote illness and cause loss of life. For example, currently more than 99% of the COVID-19 deaths now occur among unvaccinated people, and almost as many hospitalizations. To begin, the following steps must be considered:

- The President of the United States, together with science leaders at the federal agencies, should prepare and deliver a robust, public, and highly visible statement of support. The statement would reaffirm the contribution of scientists across United States history.
- We should look at expanded protection mechanisms for scientists currently targeted by far-right extremism in the United States. Rep. Paul Tonko (D-NY) has introduced a bill known as the Scientific Integrity Act of 2021 (H.R. 849) to protect US Government scientists from political interference, but this needs to be extended for scientists at private research universities and institutes. Still another possibility is to extend federal hate-crime protections.

As Nobel Laureate and Holocaust survivor Elie Wiesel once pointed out, neutrality or silence favors the oppressor. We must take steps to protect our scientists and take swift and positive action to counter the growing wave of far-right antiscience aggression. Not taking action is a tacit endorsement, and a guarantee that the integrity and productivity of science in the United States will be eroded or lose ground.
